# Adjuvant chemotherapy for undifferentiated embryonal sarcoma of the liver in adults: a case report and literature review

**DOI:** 10.3389/fonc.2025.1709059

**Published:** 2026-01-07

**Authors:** Miaogang Xiong, Zheng Li, Yang Xiang, Gang Yang, Yongfu Xiong

**Affiliations:** National Key Clinical Specialty - General Surgery, Hepatobiliary Surgery, Academician (Expert) Workstation, Sichuan Digestive System Disease Clinical Medical Research Center, Affiliated Hospital of North Sichuan Medical College, Nanchong, China

**Keywords:** adjuvant chemotherapy, adult, hepatectomy, prognosis, recurrence, undifferentiated embryonal sarcoma of the liver

## Abstract

Undifferentiated embryonal sarcoma of the liver (UESL) is a rare, highly aggressive malignant tumor that occurs predominantly in children and is exceedingly uncommon in adults. Its clinical presentation and auxiliary examinations lack specificity, making preoperative diagnosis challenging and the misdiagnosis rate high. This article reports a 33-year-old female adult with UESL who presented with right upper quadrant pain. MRI revealed a large mass in the right hepatic lobe. The patient underwent laparoscopic right hemihepatectomy and was diagnosed postoperatively. She subsequently received doxorubicin-based adjuvant chemotherapy. The disease recurred one year after surgery. Despite further surgical intervention and comprehensive treatment, the patient died 24 months postoperatively. This case suggests that while R0 resection combined with anthracycline-based adjuvant chemotherapy can prolong progression-free survival, adult UESL still carries a high risk of early recurrence and has a poor prognosis. After recurrence, the disease often progresses explosively and is resistant to multiple treatment modalities. By presenting the diagnosis and treatment experience and lessons from failure in this case, this study provides valuable insights for the clinical management of this rare disease.

## Introduction

Undifferentiated embryonal sarcoma of the liver (UESL) was first described by Stocker and Ishak in 1978 (1). It occurs mostly in children and is extremely rare in adults, accounting for less than 1% of all primary liver tumors in adults (2). A recent systematic review and pooled analysis indicated that fewer than 90 cases of adult UESL were diagnosed worldwide between 1973 and 2019 (3). Its pathogenesis remains incompletely understood; current research suggests that TP53 gene mutations may be involved in the malignant transformation of mesenchymal hamartoma (4). Historically, patients treated with surgery alone had an extremely poor prognosis, with a median survival often less than one year (1, 5). Currently, a multimodal treatment strategy incorporating R0 resection and adjuvant chemotherapy can significantly improve both progression-free survival and overall survival (3, 6–8). However, the biological behavior of adult UESL may be more aggressive than that in children (3), and its sensitivity to standard chemotherapy regimens and long-term prognosis remain suboptimal, indicating a need for further optimization of treatment strategies. This article reports the treatment course and outcome of an adult UESL case to illustrate the value of multimodal therapy while focusing on analyzing the reasons for early recurrence and therapeutic difficulties, aiming to provide an in-depth discussion of its recurrence risk and associated clinical challenges.

## Case presentation

**Figure 1:**
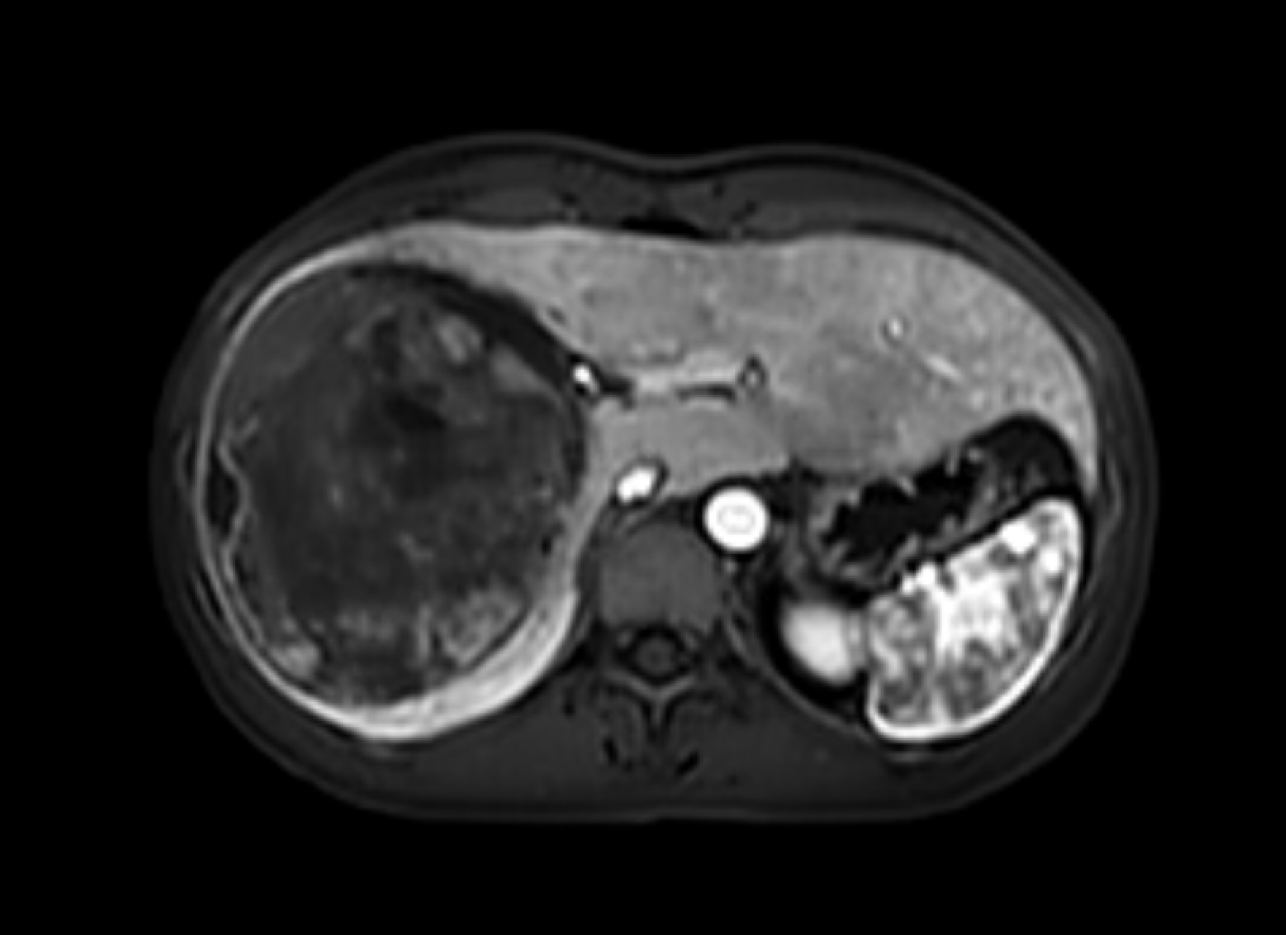
A large, round-like mass is visible in the right lobe of the liver, measuring approximately 10.9 × 9.6 cm, demonstrating abnormal signal intensity. The mass margin is relatively well-defined, and a capsule can be observed.

A 33-year-old woman was admitted to the hospital due to severe paroxysmal pain in the right upper abdomen for 7 days, accompanied by nausea and vomiting. Laboratory tests revealed elevated levels of neutrophils, alkaline phosphatase, and high-sensitivity C-reactive protein (neutrophils 7.46, normal range 1.4–7.1 × 10^9^/L; alkaline phosphatase 284, normal range 35–100 U/L; high-sensitivity C-reactive protein 206.06, normal range 0–9 mg/L). AST and ALT levels were within normal limits. Hepatitis serology results were negative. No elevation was observed in tumor markers such as alpha-fetoprotein, abnormal prothrombin, or carcinoembryonic antigen. Abdominal MRI indicated a large, round-like abnormal signal focus in the right hepatic lobe, measuring approximately 10.9 × 9.6 cm, with relatively clear boundaries and a visible capsule. On T1-weighted imaging, the lesion showed iso- to slightly high signal intensity. On T2-weighted imaging, it exhibited mixed iso-, low-, and slightly high signal intensity. Diffusion-weighted imaging sequences demonstrated restricted diffusion. Contrast-enhanced scanning revealed that the solid components primarily showed delayed enhancement. Adjacent intrahepatic bile ducts were slightly compressed and dilated. A periportal halo sign was observed around the branches of the intrahepatic portal vein. (**Figure 1**).

**Figure 2:**
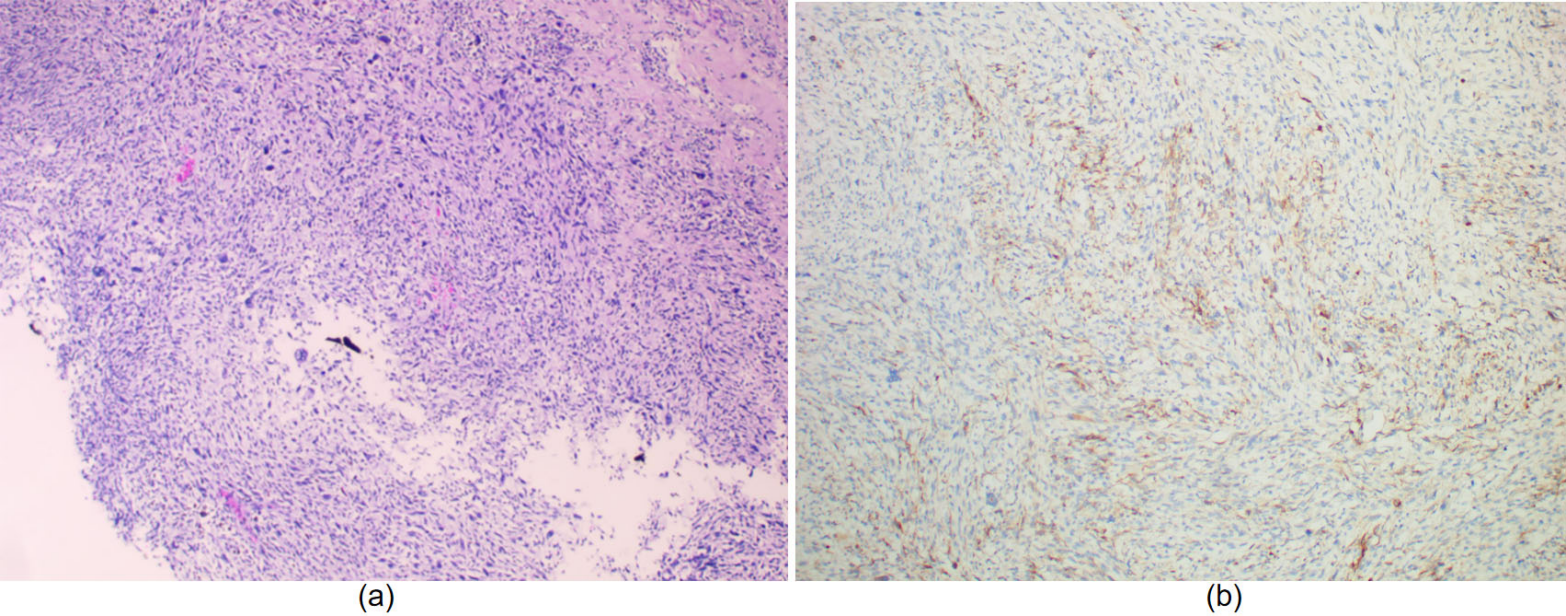
**(a)** The tumor shows significantly increased cellular density, predominantly composed of spindle cells arranged in a fibrosarcoma-like growth pattern, with visible mitotic figures.; **(b)**. Immunohistochemical staining showing focal positivity for GPC-3.

The initial diagnosis was focal nodular hyperplasia of the liver. The patient underwent laparoscopic right hemihepatectomy, and intraoperative confirmation of R0 resection was achieved. Pathological examination of the resected specimen indicated a malignant tumor in the right hepatic lobe with hemorrhage and necrosis, without involvement of the resection margin or liver capsule (**Figure 2a**).

**Figure 3:**
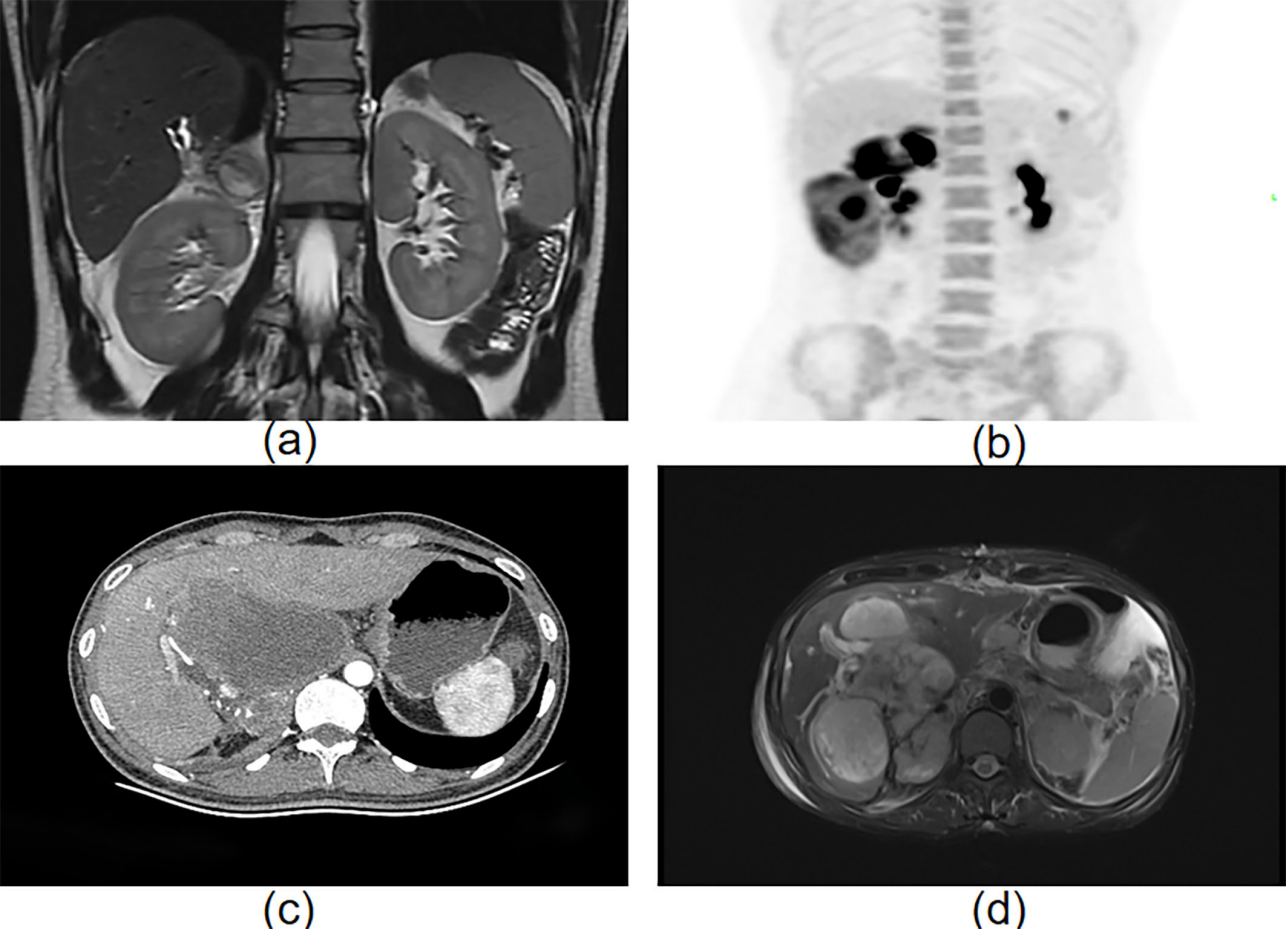
**(a)** A round-like signal shadow, measuring approximately 3.2 cm × 3.5 cm, is seen near the right adrenal area below the surgical bed; **(b)** Multiple nodular and mass-like opacities with abnormally elevated glucose metabolism are observed in the right adrenal region, right paracolic gutter, below the left lateral lobe of the liver, and the left anterior diaphragm; **(c)** Irregular masses and nodular lesions of heterogeneous density are noted in the subhepatic area, anterior right renal space, hepatic portal area, hepatorenal recess, and right adrenal area; **(d)** Multiple nodular and mass-like abnormal signal foci are present within the liver, with the largest lesion measuring approximately 4.6 cm × 6.6 cm × 5.3 cm.

Immunohistochemical results supported the diagnosis of UESL: GPC-3 (+, focal) (**Figure 2b**). EMA (–), CK (–), CK7 (–), S100 (–), SMA (–), desmin (–), Dog-1 (–), CD21 (–), and Myogenin (–) were all negative. Immunohistochemical analysis revealed no abnormal overexpression of p53. The Ki-67 proliferation index was approximately 40%. Two months after surgery, the patient began standard adjuvant chemotherapy (doxorubicin 40 mg/m² + cyclophosphamide 800 mg/m² + mesna, every three weeks for six cycles), which was well tolerated. One year after surgery, follow-up MRI revealed local recurrence (**Figure 3a**), and resection of the recurrent lesion confirmed the diagnosis. Subsequent treatment was adjusted to: gemcitabine 1000 mg/m² d1,8 + anlotinib 10 mg qd d1–14 + tislelizumab 200 mg d1, every 3 weeks, followed by maintenance therapy with tislelizumab. During maintenance therapy, PET-CT follow-up revealed multiple abdominal metastases (**Figure 3b**). To control local disease progression, palliative radiotherapy was administered to the lesions. Unfortunately, the tumor continued to progress, with one abdominal mass enlarging and compressing the duodenum (**Figure 3c**), resulting in complete obstruction and the inability to eat orally. To relieve obstruction and alleviate symptoms, palliative debulking surgery was performed. Only one month after debulking surgery, follow-up CT showed new multiple metastatic foci in the liver, the largest measuring approximately 6.6 cm in diameter (**Figure 3d**), indicating explosive disease progression. The patient died shortly thereafter.

## Discussion

Through R0 resection combined with anthracycline-based adjuvant chemotherapy, this patient achieved a progression-free survival of 12 months. This strongly supports the current view that R0 resection combined with anthracycline-based adjuvant chemotherapy is a key strategy for improving the prognosis of adult UESL. However, the disease still recurred 12 months postoperatively, ultimately leading to the patient’s death. This clinical outcome highlights the therapeutic challenges of this disease in adult patients and prompts a re-evaluation of the limitations of current treatment strategies.

UESL is a rare and highly aggressive mesenchymal tumor that predominantly occurs in older children (9). Its clinical manifestations lack specificity, primarily comprising abdominal pain or discomfort, fever, and an abdominal mass; other symptoms and signs include weight loss, anorexia, nausea, vomiting, and jaundice (3). Radiologically, ultrasonography typically reveals a large mass with mixed solid and cystic components (6). CT often demonstrates a large, low-density mass with multiple septations (6). MRI is useful for surgical planning as it can detect vascular invasion, biliary obstruction, and hilar lymphadenopathy; however, it lacks diagnostic specificity, as these imaging features are also commonly seen in various benign and malignant conditions such as hepatic hydatid cyst, abscess, and mesenchymal hamartoma (10). Laboratory investigations, including liver function tests, tumor markers, and complete blood count, are usually within normal ranges (6). Consequently, preoperative clinical diagnosis is exceptionally difficult and carries a high rate of misdiagnosis. In the case presented here, systematic evaluation initially led to a working diagnosis of focal nodular hyperplasia of the liver. When encountering a solitary large intrahepatic mass with normal tumor markers and no history of hepatitis or alcohol abuse, UESL should be considered. Histopathology remains the gold standard for definitive diagnosis. Preoperative biopsy requires careful assessment due to the tumor’s fragile texture and the risk of rupture and seeding (11). Immunohistochemistry is employed for differential diagnosis: UESL exhibits positive reactivity for vimentin, desmin, CD68, B-cell lymphoma 2, and α1-antitrypsin, while showing negative reactivity for HepPar-1, adhesion molecule, CD34, C-kit (CD117), surfactant, anaplastic lymphoma kinase, and S100 (6).

Historical data indicate that UESL patients treated with surgery alone had a median survival of less than one year and an extremely poor prognosis (1, 5). In a systematic review and pooled analysis of 308 patients, the 5-year overall survival rate was 65.8% (3). Although the recurrence at one year in this case represents an improvement compared to historical data, highlighting the role of adjuvant chemotherapy, it still underscores the high recurrence risk of UESL. A National Cancer Database study showed that the 5-year overall survival of adult UESL patients (48.2%) was significantly lower than that of pediatric patients (84.4%) (8), suggesting that adult UESL may be more aggressive or less responsive to standard chemotherapy. The patient in this case initially received adjuvant chemotherapy with doxorubicin combined with cyclophosphamide (the doxorubicin-cyclophosphamide regimen). This choice was primarily based on previous limited reports in adult cases, which suggested that regimens containing doxorubicin might be effective (12, 13). However, the treatment failure in this case indicates that the standard doxorubicin-cyclophosphamide regimen may be insufficient for adult UESL. Recent literature suggests that, unlike the vincristine, actinomycin D, and cyclophosphamide regimen commonly used in pediatric patients, chemotherapy regimens containing ifosfamide (such as the ifosfamide, vincristine, and actinomycin D regimen) may demonstrate better efficacy in adult UESL (4, 10, 14, 15). This may be due to ifosfamide’s stronger antitumor activity against certain sarcoma subtypes. Therefore, the adjuvant chemotherapy regimen for adult UESL urgently requires optimization; future approaches should consider drawing from the chemotherapy experience for soft tissue sarcomas or exploring more individualized regimens based on molecular characteristics. However, due to the extreme rarity of this disease, high-level evidence is currently lacking to establish a standard treatment protocol. We summarized previously reported cases in **Table 1**. A descriptive analysis of the cases in **Table 1** showed that the recurrence rate was lower in those who received adjuvant chemotherapy than in those who did not (16.7% [3/18] vs. 55.6% [5/9]), and mortality was higher among patients with recurrence (71.4% [5/7] vs. 6.3% [1/16]). As this is a summary of rare cases, formal statistical testing was not performed, but this trend suggests that adjuvant chemotherapy may reduce the risk of recurrence, and recurrence is a key factor influencing prognosis.

**Table 1:** Chemotherapy cases post-surgery for adult UESL.

Reference	Year	Age/Sex	Tumor sizes (cm)	Surgery	Adjuvant chemotherapy	Recurrence(treatment)	Follow up (months)
Kanamaru (12)	1991	21/F	18×15×13	Left hepatectomy	DOX CIS VIN DZN	None	12
Reichel (16)	1994	26/F	18×10×11	Lobectomy	EPI-DOX IFO	11; RT and chemotherapy	24; Deceased
Tokunaga (17)	2000	27/M	9×9×8	Bisegmentectomy (segments 5 and 6) of the liver	IFO DOX CIS	None	14
Yedibela (18)	2000	29/F	18×15×14	Trisegmentectomy	VIN IFO MES DOX	None	6
Shufaro (19)	2002	27/F	N/A	Right hepatectomy	VIN ACT CYC CIS DOX	18; RT, chemotherapy and second hepatectomy	33
Almogy (20)	2004	25/F	14	Right hepatectomy	VIN ACT CYC	Yes; RT, ifosfamide-based systemic Chemotherapy and second hepatectomy	60
Almogy (20)	2004	19/F	20	Trisegmentectomy	IFO DOX	None	47
Lepreux (21)	2005	18/F	26×21×20	A large surgical excision of the right lobe including segments IV and I	VIN DOX IFO	Yes; chemotherapy and second hepatectomy	76; Deceased
Pachera (22)	2008	22/F	11×14×19	Right trisectionectomy with extrahepatic bile duct resection reconstruction was performed	VIN ACT CYC	None	14
Faraj (23)	2010	21/M	22×19×23.6	Extended right hepatectomy	IFO ETP ACT VIN	None	5
Gasljevic (24)	2011	58/F	10	2 liver segments, the gallbladder and a part of the duodenum were resected	CYS 5-FU ONC	None	10; Deceased
Cao (13)	2014	24/F	N/A	Lobectomy	MES DOX IPH DZN	4	24
Giakoustidis (25)	2016	29/F	18	Right trisectionectomy	CYC CIS DOX	12; chemotherapy	28; Deceased
Pinamonti (26)	2018	60/F	15×12×23	En bloc resection of the mass and a S5-S6 liver bisegmentectomy	VIN ACT CYC	None	30
Beksac (27)	2018	26/F	17×12×17	Nonanatomic liver, cholecystectomy and extrahepatic biliary tract resection	PAC CIS IFO MES	None	72
Capozza (10)	2019	20/F	15×9	Right hepatectomy	VIN ACT IFO DOX	None	168
Perl (14)	2020	46/M	5.3×6	Section of liver segment VII	ACT IFO VIN	None	12
Shu (5)	2020	24/F	20×15×12.5	Extended left hemihepatectomy	DZN LOB HAIC(EPI- RUB/OXA)	Yes	17; Deceased
Shimagaki (4)	2022	20/F	9×6.5×4.5	Right hemi-hepatectomy	IFO ETP	None	12
Marques (28)	2023	49/F	18×12×20	Right hepatectomy	VIN ACT CYC	None	51
García (15)	2024	19/F	13×8×5	in Segments 6 and 7 of the liver	DOX IFO	None	34

Adri, Adriamycin; Act, Actinomycin D; Cis, Cisplatin; Cyc, Cyclophosphamide; Cys, Cysplatinum; Dzn, Dacarbazine; Dox, Doxoru-bicin; Epi-dox, Epidoxorubicin; Epi-rub, Epirubicin; Etp, Etoposide; 5-FU, 5-fluorouracil; Ifo, Ifosfamide; Iph, Iphosph-amide; Lob, Lobaplatin; Mes, Mesna; Onc, Oncovin; Oxa, Oxaliplatin; Pac, Paclitaxel; Vin, Vincristine; RT, RadiationTherapy; HAIC, Hepatic Artery Infusion Chemotherapy.

R0 resection and adjuvant chemotherapy provided the patient with a valuable 12-month disease-free period. It is noteworthy that, despite achieving a 12-month disease-free survival through R0 resection and adjuvant chemotherapy, the disease still recurred rapidly within a short timeframe. This observation prompts us to consider: should more potent chemotherapy regimens be adopted during the initial treatment phase? Is there a need for risk stratification in adult UESL to implement individualized treatment strategies for patients with different risk profiles? These questions urgently require support from more clinical data. However, after recurrence, despite attempts with comprehensive measures including reoperation, palliative radiotherapy, and a combination of anti-angiogenic drugs with immune checkpoint inhibitors, the tumor continued to progress explosively. Of greater concern is the therapeutic dilemma post-recurrence. Based on the important role of tumor angiogenesis in sarcoma progression and the significant efficacy of immunotherapy in some solid tumors, we attempted a triple-drug regimen of gemcitabine combined with anlotinib and tislelizumab. Among these, anlotinib, as a multi-target anti-angiogenic drug, theoretically inhibits tumor angiogenesis by targeting VEGFR, PDGFR, among others; anti-angiogenic drugs have been proven to improve the tumor immune microenvironment and may synergize with PD-1 inhibitors. However, this regimen failed to curb disease progression, which may reflect the high heterogeneity and complexity of UESL. We speculate that the tumor might harbor other, yet unidentified, resistance mechanisms, or its immune microenvironment characteristics may be unfavorable for immunotherapy efficacy. Particularly, the emergence of new intrahepatic metastatic foci merely one month after debulking surgery fully demonstrates the highly aggressive nature and strong metastatic potential of recurrent UESL in adults. Current treatment strategies show extremely limited effectiveness against recurrent/metastatic UESL. This failure experience highlights that for rare tumors like UESL, greater emphasis should be placed on molecular characterization in the future. Utilizing technologies such as whole-exome sequencing and RNA sequencing may identify targetable genetic alterations like NTRK fusions or ALK rearrangements, providing direction for precision therapy. Simultaneously, establishing an international case registry system to collect more clinical and molecular biological data is crucial for advancing research on this disease.

This study has several limitations. First and foremost, it is a single-case report. Although we have incorporated a literature review into the discussion, the experience and conclusions from a single case cannot represent all adult UESL patients, and the level of evidence is limited. Secondly, due to the extreme rarity of the disease, we were unable to provide data from a large patient cohort from our institution or from a prospectively designed study, which limits our ability to perform statistical analysis or draw more generalizable conclusions. Thirdly, this study is retrospective; some clinical or laboratory data, such as more comprehensive molecular marker testing, may not have been systematically collected. Finally, despite attempting multiple treatment strategies, we were unable to perform in-depth molecular analyses, such as next-generation sequencing, on the recurrent tumor, and thus failed to elucidate the specific mechanisms underlying its drug resistance. These limitations highlight the urgent need to study this rare disease through multicenter collaboration and the establishment of an international registry system.

In summary, R0 resection combined with anthracycline-based chemotherapy remains the foundational treatment for adult UESL, yet it is associated with a high risk of early recurrence, often followed by aggressive and refractory progression. Future efforts should focus on optimizing adjuvant regimens, and elucidating the molecular profile of UESL to identify potential therapeutic targets. Moreover, establishing multicenter collaborations is essential to generate robust clinical evidence. Ultimately, improving outcomes for this rare disease will require multidisciplinary research to develop more effective strategies for both initial and recurrent/metastatic settings.

## Data Availability

The original contributions presented in the study are included in the article/supplementary material. Further inquiries can be directed to the corresponding author.
