# Correction: Adjuvant chemotherapy for undifferentiated embryonal sarcoma of the liver in adults: a case report and literature review

**DOI:** 10.3389/fonc.2026.1783797

**Published:** 2026-02-04

**Authors:** Miaogang Xiong, Zheng Li, Yang Xiang, Gang Yang, Yongfu Xiong

**Affiliations:** National Key Clinical Specialty - General Surgery, Hepatobiliary Surgery, Academician (Expert) Workstation, Sichuan Digestive System Disease Clinical Medical Research Center, Affiliated Hospital of North Sichuan Medical College, Nanchong, China

**Keywords:** adjuvant chemotherapy, adult, hepatectomy, prognosis, recurrence, undifferentiated embryonal sarcoma of the liver

Affiliation “National Key Clinical Specialty - General Surgery, Hepatobiliary Surgery, Academician (Expert) Workstation, Sichuan Digestive System Disease Clinical Medical Research Center, Affiliated Hospital of North Sichuan Medical College, Nanchong, China.” was erroneously given as “^1^General Surgery Department, Affiliated Hospital of North Sichuan Medical College, Nanchong, China and ^2^North Sichuan Medical College, Nanchong, China”.

The original version of this article has been updated.

An incorrect **Funding** statement was provided. The correct funder is

“1. Noncommunicable Chronic Diseases-National Science and Technology Major Project (grant number 2023ZD0500700).

2. Innovative Team Project of Affiliated Hospital of North Sichuan Medical College, (grant number 2024CX0026).

3. Oncology Special Project of Sichuan Medical Association Sponsored by Sichuan Provincial Health Commission, (grant number 2024HR03).

4. Basic Research Platform Project of Nanchong Science and Technology Bureau, (grant number 23JCYJPT0043).

5. China Postdoctoral Science Foundation, (grant number 2023MD734156).

6. General Program of Open Research Fund of State Key Laboratory of Neuro-Oncology Drug Research and Development, (grant number SKLSIM-20251026).

7. Joint Research Project for the Development of the Affiliated Hospital of North Sichuan Medical College and Guang’an District People’s Hospital in 2025, (grant number 2025LHFZ04).”

The correct funding statement reads:

“The author(s) declared that financial support was received for this work and/or its publication. This work was supported by: 1. Noncommunicable Chronic Diseases-National Science and Technology Major Project (grant number 2023ZD0500700). 2. Innovative Team Project of Affiliated Hospital of North Sichuan Medical College (grant number 2024CX0026). 3. Oncology Special Project of Sichuan Medical Association Sponsored by Sichuan Provincial Health Commission (grant number 2024HR03). 4. Basic Research Platform Project of Nanchong Science and Technology Bureau (grant number 23JCYJPT0043). 5. China Postdoctoral Science Foundation (grant number 2023MD734156). 6. General Program of Open Research Fund of State Key Laboratory of Neuro-Oncology Drug Research and Development (grant number SKLSIM-20251026). 7. Joint Research Project for the Development of the Affiliated Hospital of North Sichuan Medical College and Guang’an District People’s Hospital in 2025 (grant number 2025LHFZ04).”

The original version of this article has been updated.

